# Association of Dietary Intake of Zinc and Selenium with Breast Cancer Risk: A Case-Control Study in Chinese Women

**DOI:** 10.3390/nu15143253

**Published:** 2023-07-22

**Authors:** Kexin Tu, Kaiyan Liu, Yifan Wang, Yiling Jiang, Caixia Zhang

**Affiliations:** 1 Department of Epidemiology, School of Public Health, Sun Yat-sen University, Guangzhou 510080, China; tukx@mail2.sysu.edu.cn (K.T.); liuky9@mail2.sysu.edu.cn (K.L.); wangyf369@mail2.sysu.edu.cn (Y.W.); jiangyling6@mail2.sysu.edu.cn (Y.J.); 2 Guangdong Provincial Key Laboratory of Food, Nutrition and Health, School of Public Health, Sun Yat-sen University, Guangzhou 510080, China

**Keywords:** zinc, selenium, breast cancer, case-control study

## Abstract

As major nonenzymatic antioxidant components in the body, dietary Zinc (Zn) and Selenium (Se) may have an impact on breast cancer development. This study aimed to investigate the relationship between dietary Zn, Se intake and breast cancer risk in Chinese women. The case-control study included 1591 cases and 1622 age-frequency matched controls. Dietary intake was collected using a validated food frequency questionnaire. Dietary Zn and Se were divided into four categories: Zn/Se from plants, Zn/Se from meat, Zn/Se from red meat, and Zn/Se from white meat. Unconditional logistic regression models and restricted cubic spline analyses were performed to identify potential associations. Zn from white meat intake was linearly and inversely associated with breast cancer risk, and Se from red meat intake was linearly and positively associated with breast cancer risk, with adjusted odds ratio and 95% confidence interval of 0.76 (0.61–0.95) and 1.36 (1.04–1.77), respectively. Non-linear relationships were found between total dietary Zn, Zn from meat, Zn from red meat intake and breast cancer risk (*p*_non-linearity_ < 0.05). In conclusion, dietary Zn and Se intake were associated with breast cancer risk in Chinese women, and the optimal intake of Zn may be beneficial for breast cancer prevention.

## 1. Introduction

Breast cancer is the most frequent malignancy in women worldwide and accounts for 30% of all newly diagnosed cancers [1]. The cancer spectrum in China has shifted towards the West in recent years, and the cancer burden of breast cancer is rising quickly [2]. About 20% of cancer incidence in developing countries can be explained by dietary-related risk factors [3].

Oxidative stress may be associated with the development of breast cancer [4,5]. As an essential trace element for humans, Zinc (Zn) may retard oxidative stress through various mechanisms, such as participating in the synthesis of Cu, Zn-superoxide dismutase, upregulating the expression of nuclear factor erythroid 2-related factor 2, and increasing the activity of glutathione peroxidase [6]. Zn deficiency may promote tumor progression by increasing the expression of nuclear factor kappa-B-dependent protumorigenic cytokines [7]. Several studies using animal models have revealed that Zn may have a preventive effect against breast cancer [8,9,10]. However, the findings of epidemiological studies investigating the relationship between dietary Zn and breast cancer risk are inconclusive [11,12,13,14,15,16].

In the human body, Selenium (Se) protects against oxidative stress primarily via some selenoenzymes, such as glutathione peroxidases, possessing powerful effects of scavenging free radicals and preventing cancer [17,18]. Selenoproteins with functional characteristics generally possess redox activities and have been found to either prevent or promote cancer [19]. Se was reported as a “dual-surface” element [20], which may have anticarcinogenic effects at low levels while having genotoxic and carcinogenic effects at doses greater than those necessary for nutrition [21,22]. In addition, foods contain different forms of Se, including selenomethionine (SeMet), selenite, selenate, selenocysteine, γ-glutamyl-selenomethionine, and Se-methyl-selenocysteine [23]. It was reported that the amounts of Se and the percentage of each form of Se to total/extractable Se varied in different kinds of foods [23]. The positive and negative effects of Se are determined by its dose and form [24]. It is still unknown how Se affects the occurrence of breast cancer [25]. Most epidemiological studies reported no significant relationship between dietary Se intake and breast cancer risk [11,13,16,26,27,28,29]; however, a few studies found a negative association [30,31,32,33].

Zn intake was found to be non-linearly associated with several other diseases [34,35,36,37,38], and there is a U-shaped curve relationship between Se levels and health risk [17,24]. So far, however, no previous study has reported the non-linear dose–response relationship between dietary Zn/Se and breast cancer risk. It was suggested that racial and ethnic differences might influence the association of Se with breast cancer risk [25]. Nevertheless, no study has been reported to be conducted among Chinese women. Furthermore, no previous study reported the relationship between Zn/Se from different food sources and breast cancer risk. Therefore, this study is intended to explore the relationship between different sources of dietary Zn, Se and breast cancer risk in Chinese women. The results will provide scientific basis for the dietary preventive strategies of breast cancer.

## 2. Materials and Methods

### 2.1. Study Population

The detailed recruitment procedures and collection of data were described elsewhere [39,40,41]. This ongoing case-control study used an age-frequency matching design. Cases were recruited from three teaching hospitals in the Guangzhou area. Eligible breast cancer patients were diagnosed between June 2007 and March 2019. Inclusion criteria included histopathology-confirmed breast cancer, 25–70 years old, natives of Guangdong or having lived in Guangdong province for at least 5 years, and the time interval between diagnosis and investigation was no more than 3 months. Exclusion criteria included having a prior history of other cancers or having a communication disorder. Controls were patients from the same hospitals’ non-oncology departments who were admitted during the same time as the cases. Controls were frequency matched to cases by age (±5 years). Inclusion criteria were similar to cases except for not being diagnosed with any cancers. Exclusion criteria included having a communication disorder. In total, we identified 1778 eligible breast cancer cases and 1786 eligible controls. Among them, 1600 cases and 1622 controls were ultimately investigated, with response rates of 89.99% and 90.82%, respectively. After excluding 9 cases with very low (<600 kcal/d) or very high (>3500 kcal/d) energy intakes [42], the final analysis included 1591 breast cancer cases and 1622 controls.

### 2.2. Data Collection

The investigators conducted face-to-face interviews after obtaining informed consent from the participants. The Women’s Health Status Questionnaire and the Lifestyle and Dietary Habits Questionnaire were used, which asked about sociodemographic information (age, education, occupation, monthly household income, etc.), lifestyle habits, dietary habits, previous disease history and family history of cancer, fertility, and menstrual history.

### 2.3. Measurement of Dietary Exposure

Investigators used the food frequency questionnaire (FFQ), which had been assessed for reliability and validity [43], to collect study subjects’ dietary intake in the previous year (one year before cases were diagnosed and one year before controls were interviewed). The FFQ contained 81 food subcategories within its 7 major categories (grains and cereals, legumes and soy products, vegetables, fruits, animal foods, mushrooms and nuts, beverages and soups). The frequency of consumption (e.g., not eaten, daily, weekly, monthly, or yearly) and serving size (e.g., medium bowl, slice, medium piece, cup, liang, medium grain, piece, small spoon, medium box, medium slice, strip, etc.) were investigated and used to calculate the average daily intake of each food group. The amounts of Zn, Se, and other nutrients in the consumed foods was calculated based on the 2002 China Food Composition Table [44]. The amount of Zn or Se in each food item was added to determine the total average daily intake of Zn and Se for the study subjects. An atlas of the illustrative food picture was used in the process of dietary survey to estimate serving sizes accurately. Dietary Zn and Se were divided into four categories: Zn/Se from plants, Zn/Se from meat, Zn/Se from red meat, and Zn/Se from white meat.

### 2.4. Statistical Analysis

Two individuals extracted all the data separately and tested for consistency. All analyses were conducted with IBM SPSS Statistics^®^ version 25 (IBM Corp., Armonk, NY, USA), Stata 15.1 (STATA Corp., College Station, TX, USA), and R version 4.2.1. Continuous variables were described by mean ± standard deviation (SD) (age, BMI, etc.) or median (25th, 75th percentile) (nutrient intake, etc.), and a *t*-test or Wilcoxon rank-sum test was used to assess differences. The number of subjects (percentage) was used to describe the categorical variables and the *χ*^2^ test was used for the equilibrium test. The natural logarithm was used to turn the dietary data into a normal distribution, and the energy residual method was used to adjust the consumption of dietary nutrients [45]. For the different sources of Zn and Se, all study participants were categorized into four classes (quartile 1 (Q1) to quartile 4 (Q4)) based on the quartile thresholds of intake for controls.

In unconditional logistic regression models, the odds ratios (ORs) and 95% confidence intervals (CIs) of the association between dietary Zn and Se intake and the risk of breast cancer were calculated for Q2–Q4, with the lowest intake group (Q1) as a reference. The following variables were considered as potential confounders based on prior research and the findings of the univariate analysis: age, education, household income, physical activity at work, ever smokers, second-hand smoke exposure, regular drinkers, BMI, family history of cancer, age at menarche, previous benign breast disease, ever use of oral contraceptives, and daily intakes of fat, fiber, vitamin A, vitamin C, and vitamin E. In regression analyses of Zn/Se from different sources, Zn/Se from plants and meat were adjusted for each other. Zn/Se from red meat and white meat were mutually adjusted, while Zn/Se from plants was adjusted concurrently. The value of *p*_trend_ was calculated by converting quartiles (Q1–Q4) to rank variables (1–4) in the logistic regression models with the minimum value as a reference. The interactions between different sources of Zn and Se were examined by including the multiplicative terms of Zn and Se (continuous variables) in the regression models. Additionally, stratified analyses were conducted according to menopausal status to investigate whether it modifies the potential association of dietary Zn/Se with breast cancer risk. The value of *p*_interaction_ was calculated by including multiplicative terms for menopausal status and dietary Zn/Se in multivariable logistic regression models.

Based on logistic regression models, restricted cubic spline (RCS) curves were employed to explore the non-linear dose–response association between dietary consumption of Zn or Se and the risk of breast cancer [46]. For different types of dietary Zn/Se, the model with the lowest Akaike information criterion (AIC) value was selected. The RCS regressions with 3 knots (at 10th, 50th, and 90th percentiles) were used to plot the non-linear relationships between total dietary Zn, Zn from plants, total dietary Se, Se from plants, Se from meat, Se from white meat, and the risk of breast cancer. RCS regressions with 4 knots (at 5th, 35th, 65th, and 95th percentiles) were used for Zn from meat, Zn from red meat, Se from red meat, and RCS regression with 5 knots (at 5th, 27.5th, 50th, 72.5th, and 95th percentiles) was used for Zn from white meat. The reference value for the RCS curve is the minimum dietary intake. To avoid the effect of outlier data on the RCS curves, study subjects with dietary intakes greater than 99% were excluded from the case and control groups, respectively. The value of *p*_non-linearity_ was calculated by the Wald test. The statistical significance level was set at *p* < 0.05 for all 2-sided tests.

## 3. Results

### 3.1. Characteristics of Cases and Controls

The analysis included data from 1591 breast cancer cases and 1622 controls. Table 1 demonstrates the characteristics of breast cancer cases and controls. The mean age of the case group was 47.79 ± 9.57 years and the mean age of the control group was 47.73 ± 9.85 years. Compared to controls, breast cancer cases had lower education levels, lower household income, lower age at menarche, but heavier physical activity at work, higher BMI, and were more likely to smoke or drink regularly, be exposed to second-hand smoke, have a family history of cancer, have a previous benign breast disease, and have a history of oral contraceptive use (*p* < 0.05). Job, age at first childbirth, breastfeeding, menopausal status, and the number of births were similar for cases and controls (*p* > 0.05).

### 3.2. Dietary Intake of Energy and Nutrients among Cases and Controls

Table 2 shows the daily intake of energy and energy-adjusted nutrients for cases and controls, including dietary Zn and Se. The median intake of energy was 1363.40 kcal/d in cases and 1355.62 kcal/d in controls. The median intakes of total dietary Zn and total dietary Se were 9.92 mg/d and 48.00 μg/d in cases and 10.21 mg/d and 49.12 μg/d in controls, respectively. Compared to controls, the intakes of total dietary Zn, Zn from plants, Zn from white meat, and total dietary Se, Se from plants, Se from white meat in cases were significantly lower (*p* < 0.05); but the intakes of Zn from red meat, Se from red meat were significantly higher (*p* < 0.05).

### 3.3. The Linear Relationship between Dietary Zn, Se and Breast Cancer Risk

As shown in Table 3, after adjusting for nondietary and dietary confounders, Zn from white meat intake was negatively associated with the risk of breast cancer. Compared to the lowest intake group (Q1), the adjusted OR (95%CI) for the highest intake group (Q4) was 0.76 (0.61–0.95, *p*_trend_ = 0.020). Total dietary Zn, Zn from plants, Zn from meat, and Zn from red meat were not statistically associated with the risk of breast cancer, with adjusted OR_Q4vsQ1_ (95%CI) of 1.06 (0.83–1.35), 0.86 (0.62–1.18), 1.11 (0.87–1.41), and 1.26 (0.99–1.60), respectively. Se from red meat intake was positively associated with the risk of breast cancer, with OR_Q4vsQ1_ (95%CI) of 1.36 (1.04–1.77, *p*_trend_ = 0.026). Total dietary Se, Se from plants, Se from meat, and Se from white meat were not statistically associated with the risk of breast cancer, with OR_Q4vsQ1_ (95%CI) of 0.86 (0.67–1.10), 0.88 (0.70–1.11), 0.93 (0.73–1.17), and 0.82 (0.66–1.02), respectively (Table 4). There was no significant interaction between different sources of Zn and Se (the values of *p*_interaction_ were not shown in the table).

### 3.4. The Non-Linear Relationship between Dietary Zn, Se, and Breast Cancer Risk

**Figure 1:**
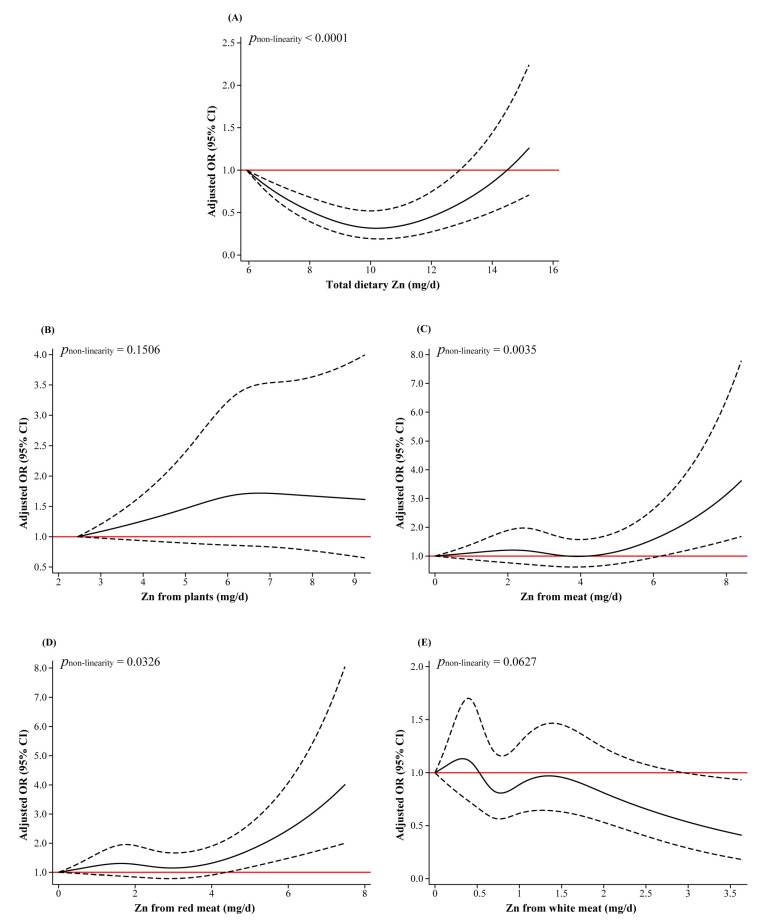
RCS plots of the non-linear dose–response relationship between dietary Zn and breast cancer. Abbreviations: OR, odds ratio; CI, confidence interval. Age, education, household income, physical activity at work, ever smokers, second-hand smoke exposure, regular drinkers, BMI, family history of cancer, age at menarche, previous benign breast disease, ever use of oral contraceptives, daily intakes of fat, fiber, vitamin A, vitamin C, and vitamin E were adjusted. In regression analyses of Zn from different sources, Zn from plants and meat were adjusted for each other. Zn from red meat and white meat were mutually adjusted, while Zn from plants was adjusted concurrently. The lowest intakes were set as references (red solid lines) (OR = 1.00). The solid line represents the OR and the dashed line represents the 95%CI of the OR.

**Figure 2:**
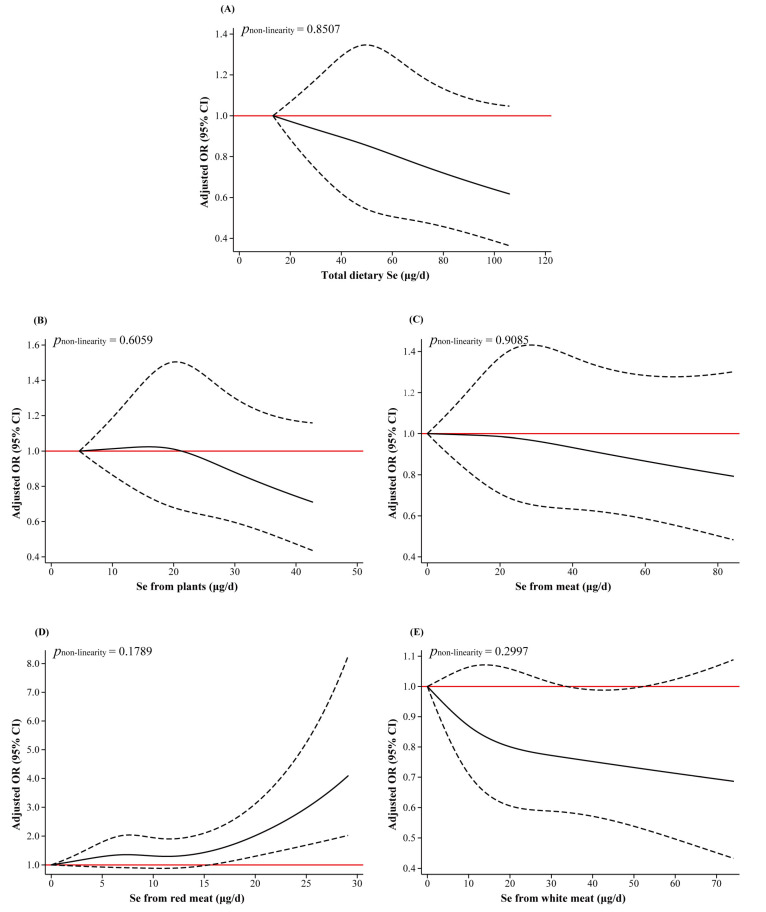
RCS plots of the non-linear dose–response relationship between dietary Se and breast cancer. Abbreviations: OR, odds ratio; CI, confidence interval. Age, education, household income, physical activity at work, ever smokers, second-hand smoke exposure, regular drinkers, BMI, family history of cancer, age at menarche, previous benign breast disease, ever use of oral contraceptives, daily intakes of fat, fiber, vitamin A, vitamin C, and vitamin E were adjusted. In regression analyses of Se from different sources, Se from plants and meat were adjusted for each other. Se from red meat and white meat were mutually adjusted, while Se from plants was adjusted concurrently. The lowest intakes were set as references (red solid lines) (OR = 1.00). The solid line represents the OR and the dashed line represents the 95%CI of the OR.

As shown in Figure 1A, there was a U-shaped non-linear relationship between total dietary Zn and breast cancer risk (*p*_non-linearity_ < 0.0001). With increasing total dietary Zn intake, the risk of breast cancer decreased first, reaching a minimum at around 10.63 mg/d, and thereafter the risk gradually increased. The RCS curve showed an inverse L-shaped association between Zn from meat intake and breast cancer risk. The risk of breast cancer increased significantly as the intake of Zn from meat exceeded 6.18 mg/d (*p*_non-linearity_ = 0.0035) (Figure 1C). Figure 1D also shows an inverse L-shaped curve for Zn from red meat intake (*p*_non-linearity_ = 0.0326), and the risk of breast cancer significantly increased when the intake exceeded 4.37 mg/d. The non-linear associations of Zn from plants intake, Zn from white meat intake with breast cancer risk were not statistically significant (*p*_non-linearity_ > 0.05) (Figure 1B,E). For dietary Se (Figure 2), no non-linear dose–response relationship was observed between different sources of Se intake and the risk of breast cancer (*p*_non-linearity_ > 0.05).

### 3.5. The Interaction between Zn, Se Intake, and Menopausal Status in Breast Cancer

**Figure 3:**
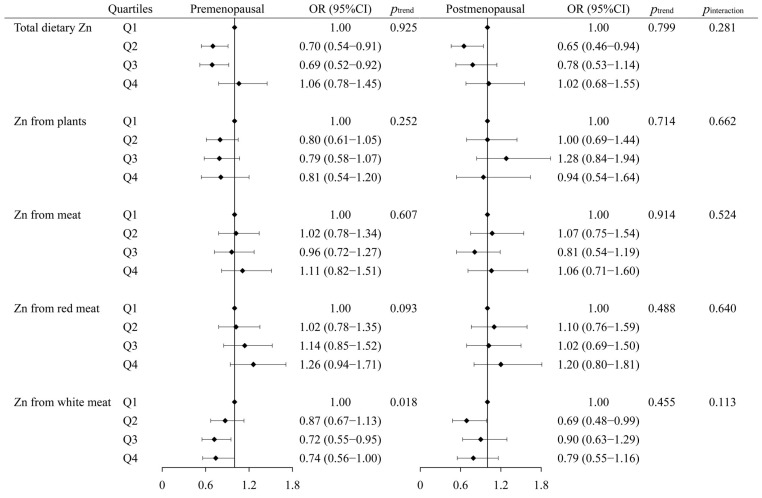
Odds ratios (ORs) and 95% confidence intervals (95%CIs) of breast cancer across quartiles of different sources of Zn according to menopausal status. The model was adjusted for age, education, household income, physical activity at work, ever smokers, second-hand smoke exposure, regular drinkers, BMI, family history of cancer, age at menarche, previous benign breast disease, ever use of oral contraceptives, daily intakes of fat, fiber, vitamin A, vitamin C, and vitamin E. In regression analyses of Zn from different sources, Zn from plants and meat were adjusted for each other. Zn from red meat and white meat were mutually adjusted, while Zn from plants was adjusted concurrently. The value of *p*_trend_ was calculated by converting quartiles (Q1–Q4) to rank variables (1–4) in models with the minimum value as a reference. *p*_interaction_ is the multiplicative interaction *p*-value between menopausal status and dietary Zn.

There were 1021 (64.17%) and 1013 (62.45%) premenopausal women in cases and controls, respectively. As shown in Figure 3, Zn from white meat intake was associated with a decreased breast cancer risk only in premenopausal women, with an adjusted OR_Q4vsQ1_ (95%CI) of 0.74 (0.56–1.00, *p*_trend_ = 0.018), but the interaction was not significant (*p*_interaction_ = 0.113). No significant association was observed between other sources of Zn intake and the risk of breast cancer in either premenopausal or postmenopausal subjects (*p*_interaction_ > 0.05).

**Figure 4:**
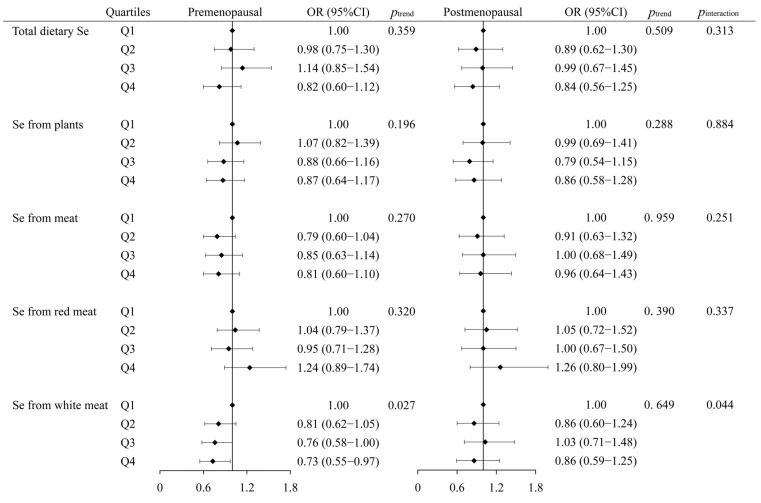
Odds ratios (ORs) and 95% confidence intervals (95%CIs) of breast cancer across quartiles of different sources of Se according to menopausal status. The model was adjusted for age, education, household income, physical activity at work, ever smokers, second-hand smoke exposure, regular drinkers, BMI, family history of cancer, age at menarche, previous benign breast disease, ever use of oral contraceptives, daily intakes of fat, fiber, vitamin A, vitamin C, and vitamin E. In regression analyses of Se from different sources, Se from plants and meat were adjusted for each other. Se from red meat and white meat were mutually adjusted, while Se from plants was adjusted concurrently. The value of *p*_trend_ was calculated by converting quartiles (Q1–Q4) to rank variables (1–4) in models with the minimum value as a reference. *p*_interaction_ is the multiplicative interaction *p*-value between menopausal status and dietary Se.

As shown in Figure 4, Se from white meat intake was associated with a decreased breast cancer risk only in premenopausal women, with an adjusted OR_Q4vsQ1_ (95%CI) of 0.73 (0.55–0.97, *p*_trend_ = 0.027), and the interaction was statistically significant (*p*_interaction_ = 0.044). No significant association was found between other sources of Se intake and the risk of breast cancer in either premenopausal or postmenopausal subjects (*p*_interaction_ > 0.05).

## 4. Discussion

In this case-control study, we aimed to investigate the relationship between dietary Zn, Se, and breast cancer risk in Chinese women. The results demonstrated a linear and negative association between Zn from white meat and breast cancer risk, and a linear and positive association between Se from red meat and breast cancer risk. According to RCS analysis, there was a significant U-shaped non-linear relationship between total dietary Zn and breast cancer risk, and an inverse L-shaped association between Zn from meat, Zn from red meat and risk. No significant associations were found between Zn from plants, total dietary Se, Se from plants, Se from meat, Se from white meat intake and breast cancer risk.

Total dietary Zn was not found to be linearly and significantly associated with breast cancer risk. Consistent with our result, some epidemiological studies did not find any evidence of the association between dietary Zn intake and breast cancer risk [13,14,15,16]. However, a small case-control study (310 cases) conducted in Germany found a negative association between dietary Zn and breast cancer risk [12]. There was a U-shaped association between total dietary Zn intake and breast cancer risk in our study, with an inflection point at about 10.63 mg/d. So far, there are no studies examining the non-linear relationship between dietary Zn and breast cancer risk. Some studies observed non-linear associations of Zn intake with other cancers and diseases [34,35,36,37,38]. For example, a U-shaped association was observed between dietary Zn intake and new-onset diabetes in Chinese adults, with an inflection point at about 9.1 mg/day [36]. Zn intake was associated with prostate cancer [34] and new-onset hypertension [35] in a J-shaped manner, whereas Zn intake showed an inverted L-shaped curve with cognitive test scores [37] and the biomarker of oxidative stress (serum malondialdehyde) [38]. The main mechanism of Zn as an anticancer factor is its antioxidant function. Additionally, the mechanisms include the effects of Zn on the immune system, cell differentiation and proliferation, DNA and RNA synthesis and repair, enzyme activation or inhibition, transcription factors, the regulation of cellular signaling, and the stabilization of the cell structure and membranes [47]. However, excess Zn intake might have a negative influence on cancer prevention due to its immunosuppressive effects [47]. Moreover, the limited margin of safety of Zn should be of some concern [48].

To date, no study has reported on the relationship between Zn from different food sources and breast cancer risk. Some studies reported that the association of dietary Zn with cardiovascular disease (CVD), metabolic syndrome (MetS), and coronary artery calcium (CAC) varied depending on its source [49,50]. Zn from red meat, rather than Zn from other sources, was positively associated with CVD and MetS risk [49], and Zn from non-red meat was independently and negatively associated with the risk of CAC progression [50]. Our study found a non-linear association of Zn from meat and red meat with the odds of breast cancer. Breast cancer risk rose when intake exceeded thresholds, 6.18 mg/day of Zn from meat and 4.37 mg/day of Zn from red meat. This association might in part reflect the role of other nutrients in meat and red meat. First of all, it can be partially attributed to other harmful carcinogenic components in meat and red meat [51]. Known and suspected human carcinogens such as N-nitroso compounds and polycyclic aromatic hydrocarbons can be formed during the processing of meat. Heterocyclic amines and polycyclic aromatic hydrocarbons can also be produced when red meat is cooked at or above 200 °C [52]. Second, heme iron from red meat was reported to be strongly correlated with Zn from red meat, with a correlation coefficient of 0.90 [49]. Thus, the association of Zn from red meat with breast cancer risk may reflect the intake of heme iron from red meat and its bioactivity [49]. Similar to the results for Zn from red meat, an inverse L-shaped association has been found between heme iron intake and breast cancer risk in this studied population [41]. Finally, the meta-analysis showed a positive association between meat or red meat intake and the risk of breast cancer [53], which may obscure the protective effect of dietary Zn.

In our study, Zn from white meat was observed to be linearly and inversely associated with breast cancer risk. It implied that the negative association of dietary Zn with breast cancer observed in previous studies may be due to the effect of Zn from white meat. The exact reasons for the inverse association are not clear due to limited literature. Further studies are warranted to verify our findings. Additionally, as the main source of dietary Zn intake in this studied population, Zn from plants was not associated with breast cancer risk.

The results of this study suggested that food sources might influence the association between Zn intake and breast cancer risk. Consequently, the inconsistent results of previous studies on the association of dietary Zn with breast cancer risk can be partially attributed to the different food sources of Zn across different populations [49]. For example, dietary Zn intake in Asian populations is derived mainly from cereals [54,55], whereas in the United States and European countries, it may be derived mainly from meat and meat products [49,56]. More research is required to further examine the association of different food sources of Zn with breast cancer risk.

Neither linear nor non-linear analyses found a significant association between total dietary Se intake and breast cancer risk. Consistent with our results, four cohort studies [16,26,27,28], two case-control studies [13,29], and a systematic review [11] did not find an association between dietary Se and breast cancer risk. On the contrary, two case-control studies from Malaysia [30,31] and one from Iran [32] suggested a negative association between dietary Se and breast cancer risk. Nonetheless, these three studies had low statistical power due to insufficient sample sizes, including 57, 64, and 145 cases, respectively. A meta-analysis including 14 studies published in 2016 assessed the association between Se exposure (dietary Se/Se supplements/serum Se/plasma Se/toenail Se) and breast cancer risk [33]. It showed that high exposure to Se effectively reduced breast cancer risk by 12%, with a combined OR (95%CI) of 0.88 (0.84–0.93). However, this meta-analysis did not individually evaluate the impact of dietary Se on breast cancer risk and lacked sufficient data for dose–response analysis. The U-shaped non-linear relationship between Se and breast cancer risk was not observed in our study, probably due to the low intake level of Se. It was noted that Se deficiency occurs when intake is below 40 μg/d, and toxicity can be observed at intakes above 400 μg/d [57]. However, the range of total dietary Se intake in RCS analysis was 13.20–105.90 μg/d, which was far below 400 μg/d.

The observed linear and positive association between Se from red meat and breast cancer risk can be partially attributed to the presence of carcinogens in red meat [52]. Additionally, it might be associated with harmful Se species. Red meat offal contains relatively high levels of Se, and the predominant species of Se in edible portions of meat may be SeMet (accounts for 50–60% of total extractable Se species) [23]. Studies reported that SeMet has toxic effects at a high dose. SeMet can be metabolized to selenol/selenate to be involved in the redox cycle, which in turn generates superoxide radicals [24,58]. It is noteworthy that some nutrients in food have interactive effects with Se that can alter the bioavailability of Se and influence the mechanisms of disease development [49]. The distribution of nutrients that interact with Se is unequal across foods, which also helps to explain the inconsistent associations of Se from different food sources with breast cancer risk. The antagonistic effect between dietary Se and arsenic, cadmium has been well demonstrated in animal models [24,59]. In cellular experiments, Se antagonized cadmium-induced breast carcinogenesis by epigenetic modifications and acted synergistically with ascorbic acid in preventing breast cancer [60,61]. In a nested case-control study, the potential association between serum iodine and breast cancer risk was modified by Se levels [62]. Therefore, although total dietary Se showed a nonsignificant negative association with breast cancer risk, Se from red meat was detrimental to breast cancer risk. This result suggests that excessive Se intake from red meat solely should be avoided.

We did not find an interaction between different sources of Zn and Se in relation to breast cancer risk. In contrast to the results of this study, a study found a significant interaction between Zn and Se intake on cognition in females [37]. The interaction between Zn and Se is currently poorly known. Cellular and molecular studies suggested a possible interaction between Zn and Se in metabolism and receptor binding [63], but such interactions have only been found in animal studies [64,65]. Further studies are needed to investigate the interactive effects of Zn and Se on human diseases.

No significant association was observed between Zn or Se intakes and breast cancer risk in either premenopausal or postmenopausal women in two previous studies [13,16]. However, these studies did not evaluate the interaction of dietary Zn or Se with menopausal status. A small cross-sectional study (65 cases) in the United States did not find a significant interaction between Se intake and menopausal status with cytologic atypia (a breast cancer risk biomarker) [66]. Higher intake of Se was associated with a lower likelihood of atypia among premenopausal women, but not among postmenopausal women. Postmenopausal women theoretically have greater benefits from antioxidant consumption than premenopausal women [67]. However, the results of our study did not support this hypothesis. The interaction between Se from white meat and menopausal status and the negative association of Zn from white meat with breast cancer risk observed only in premenopausal women may be related to the different oxidative stress statuses. A study suggested that Zn/Se consumption may act on breast tissue by affecting the expression of certain inflammation markers among premenopausal or postmenopausal women [68]. More studies are needed to illustrate the exact mechanism of the interactive effect.

This study has several strengths. First, this is the first study to assess the relationship between Zn, Se intake and breast cancer risk among Chinese women. Then, the sample size was large enough to provide sufficient statistical power to detect convincing associations. The large sample size also allowed us to conduct stratified analysis by menopausal status. Furthermore, our study examined the associations between different sources of Zn or Se intake and breast cancer risk, which provides new insights into the relationship between nutrients and cancer. Finally, the RCS regression models were used to analyze the shape of the dose–response relationship, which allowed us to observe how breast cancer risk varies at different levels of dietary Zn/Se intake [69,70].

This study also has several limitations. First of all, it can be challenging to avoid selection bias in case-control studies. However, for this study, patients with breast cancer were recruited from three large teaching hospitals within the Guangzhou area. To some extent, these cases might be representative of the patients in Guangdong province. The high response rate (89.99% for cases and 90.82% for controls) in this study also helped to reduce the impact of selection bias on the results. Second, in dietary surveys, study participants were asked to recall and report the kind and frequency of food intake in the previous year, which might be subject to recall bias. However, only new cases (diagnosed no more than 3 months before the interview) were included in this study, and an atlas of the illustrative food picture was provided to help subjects recall diet more accurately. Third, measurement errors and misclassification were also inevitable. This nondifferential misclassification might result in a dilution of the association between dietary Zn, Se intake and breast cancer risk in our study. Fourth, the subjects in this study may not be representative of women across the country. China Nutritional Transition Cohort Survey 2015 [55] showed that the average dietary Zn intake for women was 9.4 mg/d, and only 67.0% of the women had a dietary Zn intake at or above the recommended nutrient intake (RNI) for women aged 18 to 49 (7.5 mg/d) [71]. However, 98.1% of the women in our study had a dietary Zn intake at or above the RNI. Therefore, our findings may be limited to Chinese women with adequate Zn intake, and further research is required to confirm these findings. Fifth, this study did not detect tissue levels of Zn and Se. Plasma/serum levels were reported to reflect exposure for several days and weeks [72,73], and there was poor agreement between serum Zn/Se and dietary intake of Zn/Se [15,28,74]. Therefore, further studies on the association of circulating levels or biomarkers of Zn/Se status, or breast tissue-specific Zn/Se levels with breast cancer risk are needed [25]. Finally, although we comprehensively collected confounding factors associated with breast cancer risk, we could not completely exclude the effect of other potential confounders.

## 5. Conclusions

Total dietary Zn, Zn from meat, and Zn from red meat intake were non-linearly associated with breast cancer risk. An optimal intake of total dietary Zn, and Zn from white meat intake may be beneficial for breast cancer prevention, whereas Se from red meat intake may be associated with an increased risk of breast cancer. The findings reveal the potential effects of different dietary sources of Zn and Se on breast cancer risk and also provide a theoretical basis for dietary prevention of breast cancer in Chinese women.

## Figures and Tables

**Table 1:** Characteristics of breast cancer cases and controls.

	Cases (*n* = 1591)	Controls (*n* = 1622)	*p* ^a^
Age (years), mean ± SD	47.79 ± 9.57	47.73 ± 9.85	0.851
Education, *n* (%)			0.001
Primary school or below	395 (24.84)	444 (27.39)	
Middle school	454 (28.55)	392 (24.18)	
High school/technical school	389 (24.47)	379 (23.38)	
Secondary technical school	199 (12.52)	187 (11.54)	
College or above	153 (9.62)	219 (13.51)	
Job, *n* (%)			0.299
White-collar	634 (39.85)	654 (40.32)	
Blue-collar	398 (25.02)	436 (26.88)	
Farmer/other	559 (35.14)	532 (32.80)	
Household income (Yuan/month), *n* (%)			0.003
<2000	306 (19.23)	248 (15.29)	
2001–5000	491 (30.86)	467 (28.79)	
5001–8000	433 (27.22)	498 (30.70)	
≥8001	361 (22.69)	409 (25.22)	
Physical activity at work, *n* (%)			0.027
Nonworking	505 (31.74)	440 (27.13)	
Sedentary	514 (32.31)	545 (33.60)	
Low	323 (20.30)	374 (23.06)	
Moderate	136 (8.55)	159 (9.80)	
Heavy	113 (7.10)	104 (6.41)	
Ever smokers, *n* (%)	18 (1.13)	8 (0.49)	0.044
Second-hand smoke exposure, *n* (%)	946 (59.46)	831 (51.23)	<0.001
Regular drinkers, *n* (%)	195 (12.26)	114 (7.03)	<0.001
BMI (kg/m^2^), mean ± SD	23.05 ± 3.38	22.56 ± 3.16	<0.001
Family history of cancer, *n* (%)	238 (14.96)	158 (9.74)	<0.001
Age at menarche (years), mean ± SD	14.52 ± 1.90	14.76 ± 1.83	<0.001
Age at first childbirth (years) ^b^, mean ± SD	25.56 ± 3.68	25.37 ± 3.59	0.146
Breastfeeding ^b^, *n* (%)	1345 (88.49)	1393 (90.16)	0.149
Previous benign breast disease, *n* (%)	608 (38.21)	371 (22.87)	<0.001
Ever use of oral contraceptives, *n* (%)	134 (8.43)	100 (6.17)	0.014
Menopausal status, *n* (%)			0.312
Premenopausal	1021 (64.17)	1013 (62.45)	
Postmenopausal	570 (35.83)	609 (37.55)	
Number of births, *n* (%)			0.323
0	51 (3.21)	57 (3.51)	
1–2	1021 (64.17)	1075 (66.28)	
≥3	519 (32.62)	490 (30.21)	

Abbreviations: SD, standard deviation; BMI, body mass index. ^a^ Differences in continuous variables were tested using the *t*-test or Wilcoxon rank-sum test, and equilibrium tests for categorical variables were performed using the *χ*^2^ test. ^b^ Among women who have given birth to a live child.

**Table 2:** Dietary intake of energy, Zn, Se and selected nutrients among breast cancer cases and controls.

	Cases (*n* = 1591)	Controls (*n* = 1622)	*p* ^b^
Energy (kcal/d) ^a^	1363.40 (1154.85, 1634.81)	1355.62 (1164.49, 1615.31)	0.455
Dietary Zn intake (mg/d) ^a^			
Total dietary Zn	9.92 (8.90, 11.18)	10.21 (9.31, 11.27)	<0.001
Zn from plants	6.09 (5.45, 6.72)	6.32 (5.68, 7.01)	<0.001
Zn from meat	3.10 (2.21, 4.29)	3.11 (2.20, 4.20)	0.555
Zn from red meat	2.23 (1.45, 3.32)	2.15 (1.36, 3.21)	0.050
Zn from white meat	0.68 (0.39, 1.05)	0.72 (0.45, 1.12)	0.006
Dietary Se intake (μg/d) ^a^			
Total dietary Se	48.00 (39.41, 58.05)	49.12 (40.43, 59.95)	0.004
Se from plants	19.28 (14.63, 24.15)	20.45 (15.64, 24.91)	<0.001
Se from meat	22.21 (14.95, 30.65)	22.16 (15.20, 30.85)	0.795
Se from red meat	9.42 (6.10, 13.82)	8.97 (5.75, 12.99)	0.003
Se from white meat	10.41 (5.46, 18.36)	11.01 (6.25, 19.38)	0.023
Total fat (g/d) ^a^	28.91 (22.32, 36.13)	28.95 (22.92, 35.22)	0.550
Dietary fiber (g/d) ^a^	8.25 (6.83, 9.87)	9.07 (7.52, 10.84)	<0.001
Vitamin A (μgRE/d) ^a^	128.69 (94.15, 171.40)	149.23 (113.72, 193.35)	<0.001
Vitamin C (mg/d) ^a^	706.58 (527.42, 938.73)	810.71 (623.94, 1026.11)	<0.001
Vitamin E (mg/d) ^a^	9.33 (7.52, 11.91)	10.38 (8.26, 13.08)	<0.001

^a^ Dietary intake of energy and nutrients was described by median (25th, 75th percentile) and nutrients were adjusted for the energy using the residual method. ^b^ Differences in dietary intake were tested using the Wilcoxon rank-sum test.

**Table 3:** Odds ratios (ORs) and 95% confidence intervals (95%CIs) of breast cancer across quartiles of different sources of Zn intake.

	Q1	Q2	Q3	Q4	*p*_trend_ ^c^
Total dietary Zn					
*N* (cases/controls)	567/405	340/406	303/406	381/405	
Median (mg/d)	8.65	9.77	10.72	12.14	
cOR (95%CI)	1.00	0.60 (0.49–0.73)	0.53 (0.44–0.65)	0.67 (0.56–0.81)	<0.001
aOR (95%CI) ^a^	1.00	0.53 (0.43–0.65)	0.71 (0.58–0.88)	1.00 (0.99–1.01)	<0.001
aOR (95%CI) ^b^	1.00	0.68 (0.55–0.84)	0.71 (0.57–0.90)	1.06 (0.83–1.35)	0.786
Zn from plants					
*N* (cases/controls)	526/405	414/406	383/405	268/406	
Median (mg/d)	5.18	5.99	6.62	7.48	
cOR (95%CI)	1.00	0.79 (0.65–0.95)	0.73 (0.60–0.88)	0.51 (0.42–0.62)	<0.001
aOR (95%CI) ^a^	1.00	0.82 (0.67–0.99)	0.77 (0.63–0.95)	0.53 (0.43–0.65)	<0.001
aOR (95%CI) ^b^	1.00	0.91 (0.73–1.13)	0.96 (0.75–1.22)	0.86 (0.62–1.18)	0.473
Zn from meat					
*N* (cases/controls)	395/405	402/406	383/406	411/405	
Median (mg/d)	1.64	2.67	3.56	5.20	
cOR (95%CI)	1.00	1.02 (0.84–1.23)	0.97 (0.79–1.18)	1.04 (0.86–1.27)	0.818
aOR (95%CI) ^a^	1.00	1.00 (0.81–1.22)	0.95 (0.77–1.17)	1.12 (0.91–1.38)	0.401
aOR (95%CI) ^b^	1.00	0.98 (0.79–1.22)	0.94 (0.75–1.18)	1.11 (0.87–1.41)	0.496
Zn from red meat					
*N* (cases/controls)	355/405	405/406	402/406	429/405	
Median (mg/d)	0.90	1.78	2.61	4.25	
cOR (95%CI)	1.00	1.14 (0.93–1.39)	1.13 (0.93–1.38)	1.21 (0.99–1.47)	0.080
aOR (95%CI) ^a^	1.00	1.16 (0.94–1.43)	1.17 (0.95–1.45)	1.40 (1.14–1.73)	0.003
aOR (95%CI) ^b^	1.00	1.07 (0.86–1.34)	1.07 (0.85–1.34)	1.26 (0.99–1.60)	0.073
Zn from white meat					
*N* (cases/controls)	475/406	382/405	379/406	355/405	
Median (mg/d)	0.27	0.58	0.88	1.52	
cOR (95%CI)	1.00	0.81 (0.67–0.98)	0.80 (0.66–0.97)	0.75 (0.62–0.91)	0.004
aOR (95%CI) ^a^	1.00	0.76 (0.62–0.93)	0.75 (0.61–0.92)	0.64 (0.52–0.78)	<0.001
aOR (95%CI) ^b^	1.00	0.78 (0.64–0.97)	0.79 (0.64–0.97)	0.76 (0.61–0.95)	0.020

Abbreviations: Q, quartile; Q1–Q4 means the lowest quartile to the highest quartile; cOR, crude odds ratio; aOR, adjusted odds ratio. ^a^ OR was adjusted for age, education, household income, physical activity at work, ever smokers, second-hand smoke exposure, regular drinkers, BMI, family history of cancer, age at menarche, previous benign breast disease, and ever use of oral contraceptives. ^b^ OR was additionally adjusted for daily intakes of fat, fiber, vitamin A, vitamin C, and vitamin E. In regression analyses of Zn from different sources, Zn from plants and meat were adjusted for each other. Zn from red meat and white meat were mutually adjusted, while Zn from plants was adjusted concurrently. ^c^ The value of *p*_trend_ was calculated by converting quartiles (Q1–Q4) to rank variables (1–4) in models with the minimum value as a reference.

**Table 4:** Odds ratios (ORs) and 95% confidence intervals (95%CIs) of breast cancer across quartiles of different sources of Se.

	Q1	Q2	Q3	Q4	*p*_trend_ ^c^
Total dietary Se					
*N* (cases/controls)	439/405	406/406	410/406	336/405	
Median (μg/d)	34.31	44.88	54.14	68.57	
cOR (95%CI)	1.00	0.92 (0.76–1.12)	0.93 (0.77–1.13)	0.77 (0.63–0.93)	0.015
aOR (95%CI) ^a^	1.00	0.91 (0.74–1.12)	0.87 (0.70–1.07)	0.71 (0.57–0.88)	0.002
aOR (95%CI) ^b^	1.00	1.00 (0.80–1.24)	1.03 (0.82–1.30)	0.86 (0.67–1.10)	0.268
Se from plants					
*N* (cases/controls)	483/405	416/406	353/406	339/405	
Median (μg/d)	12.63	18.15	22.46	28.53	
cOR (95%CI)	1.00	0.86 (0.71–1.04)	0.73 (0.60–0.89)	0.70 (0.58–0.85)	<0.001
aOR (95%CI) ^a^	1.00	0.87 (0.71–1.07)	0.71 (0.58–0.88)	0.67 (0.54–0.83)	<0.001
aOR (95%CI) ^b^	1.00	1.01 (0.82–1.25)	0.87 (0.70–1.08)	0.88 (0.70–1.11)	0.155
Se from meat					
*N* (cases/controls)	415/405	375/406	405/406	396/405	
Median (μg/d)	11.23	18.86	25.76	40.43	
cOR (95%CI)	1.00	0.90 (0.74–1.10)	0.97 (0.80–1.18)	0.95 (0.79–1.16)	0.830
aOR (95%CI) ^a^	1.00	0.86 (0.70–1.06)	0.95 (0.77–1.17)	0.92 (0.74–1.13)	0.625
aOR (95%CI) ^b^	1.00	0.84 (0.68–1.05)	0.93 (0.74–1.18)	0.93 (0.73–1.17)	0.789
Se from red meat					
*N* (cases/controls)	357/405	373/406	400/406	461/405	
Median (μg/d)	3.78	7.40	10.66	16.55	
cOR (95%CI)	1.00	1.04 (0.85–1.27)	1.12 (0.92–1.36)	1.29 (1.06–1.57)	0.007
aOR (95%CI) ^a^	1.00	1.09 (0.88–1.34)	1.16 (0.94–1.44)	1.45 (1.18–1.79)	<0.001
aOR (95%CI) ^b^	1.00	1.02 (0.82–1.27)	1.08 (0.86–1.37)	1.36 (1.04–1.77)	0.026
Se from white meat					
*N* (cases/controls)	465/405	372/406	389/406	365/405	
Median (μg/d)	3.61	8.54	14.50	29.27	
cOR (95%CI)	1.00	0.80 (0.66–0.97)	0.83 (0.69–1.01)	0.79 (0.65–0.95)	0.025
aOR (95%CI) ^a^	1.00	0.78 (0.63–0.95)	0.76 (0.62–0.94)	0.70 (0.57–0.86)	0.001
aOR (95%CI) ^b^	1.00	0.82 (0.66–1.01)	0.85 (0.69–1.05)	0.82 (0.66–1.02)	0.108

Abbreviations: Q, quartile; Q1–Q4 means the lowest quartile to the highest quartile; cOR, crude odds ratio; aOR, adjusted odds ratio. ^a^ OR was adjusted for age, education, household income, physical activity at work, ever smokers, second-hand smoke exposure, regular drinkers, BMI, family history of cancer, age at menarche, previous benign breast disease, and ever use of oral contraceptives. ^b^ OR was additionally adjusted for daily intakes of fat, fiber, vitamin A, vitamin C, and vitamin E. In regression analyses of Se from different sources, Se from plants and meat were adjusted for each other. Se from red meat and white meat were mutually adjusted, while Se from plants was adjusted concurrently. ^c^ The value of *p*_trend_ was calculated by converting quartiles (Q1–Q4) to rank variables (1–4) in models with the minimum value as a reference.

## Data Availability

The data that support the findings of our study are available from the corresponding author upon reasonable request.
